# O4.8. VULNERABLE PERIODS FOR COGNITIVE DEVELOPMENT IN INDIVIDUALS AT HIGH GENOMIC RISK OF SCHIZOPHRENIA

**DOI:** 10.1093/schbul/sby015.214

**Published:** 2018-04-01

**Authors:** Sinead Morrison, Samuel Chawner, Therese van Amelsvoort, Ann Swillen, Elfi Vergaelen, Michael Owen, Marianne van den Bree

**Affiliations:** 1 Cardiff University; 2 Maastricht University; 3 KU Leuven

## Abstract

**Background:**

22q11.2 Deletion Syndrome (22q11.2DS) is caused by the deletion of approximately 60 genes on chromosome 22 and represents one of the strongest known genetic risk factors for schizophrenia. Approximately 1 in 4 adults with 22q11.2DS are diagnosed with schizophrenia spectrum disorders, presenting with psychotic symptomatology analogous to that exhibited in idiopathic schizophrenia.

Cognitive deficits are a core feature of schizophrenia. 22q11.2DS presents a valuable model for understanding vulnerable periods of cognitive development which may be associated with psychosis development. Most previous studies report greater deficits in older individuals with 22q11.2DS than younger individuals but these studies have often focused solely on IQ, neglecting other neurocognitive domains associated with schizophrenia. Additionally, many studies of 22q11.2DS have not included adults, missing a crucial group at increased risk for schizophrenia. The first aim was therefore to examine whether there are increasing deficits in cognitive functioning on a wide range of domains in 22q11.2DS across developmental stages (children, adolescents and adults) compared to typically developing (TD) controls. The second aim was to take into account the presence of a psychotic disorder, and whether this explained variance in functioning.

**Methods:**

We conducted the largest study to date of neurocognitive functioning beyond IQ in 22q11.2DS. This work was the result of international collaboration across 3 sites. The same battery of tasks measuring processing speed, attention and spatial working memory were completed by 219 participants with 22q11.2DS and 107 TD controls. Wechsler IQ tests were completed, yielding Full Scale (FSIQ), Verbal (VIQ) and Performance IQ scores (PIQ). An age-standardised difference score was produced for each participant taking into account TD control performance. The average performance of children (6–10 years), adolescents (10–18 years) and adults (18–56 years) was compared using an ANOVA approach. No children or adolescents reached diagnostic criteria for a psychotic disorder, but 13% of adults with 22q11.2DS were either diagnosed with a DSM-IV psychotic disorder. The cognitive performance of adults with or without a psychotic disorder was compared with independent t-tests with correction for unequal variance.

**Results:**

Children and adults with 22q11.2DS displayed a greater deficit in working memory than adolescents (p=0.017 and p<0.001 respectively). Adults displayed greater deficits in FSIQ and PIQ than adolescents (p=0.018 and p=0.001 respectively). Adults diagnosed with a psychotic disorder displayed a greater deficit in VIQ than those without a psychotic disorder (p=0.040).

**Discussion:**

Magnitude of cognitive deficit in individuals with 22q11.2DS varied by cognitive domain and developmental stage. There were specific deficits in working memory, PIQ and FSIQ in adults with 22q11.2DS compared to children and adolescents. The lack of differences between children and adolescents contradicts previous research which proposes that older children exhibit greater cognitive deficits, and suggests that there may be a longer developmental window to intervene and maintain cognitive functioning in a group at high genomic risk of schizophrenia. Adults with 22q11.2DS and psychotic disorder had a greater deficit in VIQ, which supports previous research. This international sample provides unique insights into cognitive functioning in 22q11.2DS across developmental stages.

